# Effects of Intradialytic Cognitive and Physical Exercise Training on Cognitive and Physical Abilities in Hemodialysis Patients: Study Protocol for a Randomized Controlled Trial

**DOI:** 10.3389/fpsyg.2022.835486

**Published:** 2022-01-25

**Authors:** Špela Bogataj, Nebojša Trajković, Maja Pajek, Jernej Pajek

**Affiliations:** ^1^Department of Nephrology, University Medical Centre Ljubljana, Ljubljana, Slovenia; ^2^Faculty of Sport and Physical Education, University of Niš, Niš, Serbia; ^3^Faculty of Sport, University of Ljubljana, Ljubljana, Slovenia

**Keywords:** cognitive functions, hemodialysis, physical activity, intradialytic training, non-pharmacological interventions

## Abstract

The prevalence of cognitive impairment in hemodialysis (HD) patients is extremely high. Despite the well-documented benefits of interventions on cognitive function, there is a widespread call for effective strategies that will show the long-term consequences in patients undergoing dialysis. The aim of this research protocol was to investigate the effect of cognitive training combined with physical exercise on cognitive function, physical performance, and frailty indicators in the HD population. We will conduct a randomized controlled intervention trial to examine the effects of a combined non-pharmacological intervention in the form of intradialytic physical exercise and intradialytic cognitive training on cognitive function, indicators of frailty, and physical performance measures in HD patients. The group of patients receiving the study intervention will be compared to the control group receiving standard HD care. The duration of the intervention will be 12 weeks. We will use sensitive instruments (cognitive domain tests) to assess cognitive functions. The primary outcome of the study at 12 weeks will be performance on the Alertness subtest of the computerized Test of Attentional Performance. Secondary study outcomes are: Performance in other domains of cognitive function (executive function, psychomotor speed, information processing efficiency, working memory, and attention), physical fitness (10 repetition sit-to-stand test, timed up and go test, handgrip strength test, spontaneous gait speed, and stork balance test), and assessment of frailty (Edmonton Frail Scale). Study outcomes will be assessed at baseline, immediately after the 12-week intervention, and 6 months after the end of the study without specific further intervention (retention effect assessment). This study will be among the first to test the synergistic effects of a uniquely designed physical exercise and cognitive training intervention on functional status in HD patients. We believe our results will contribute to dementia prevention research by demonstrating the long-term efficacy of our combined intervention.

**Clinical Trial Registration:** ClinicalTrials.Gov, NCT05150444.

## Introduction

The number of older adults is increasing worldwide, as mortality at younger ages is decreasing (World Report on Ageing and Health, 2015). In addition, 50 million people suffer from dementia, and experts predict that this number will increase to 152 million by 2050 (Patterson, 2018). Dementia affects not only the sufferers themselves, but also their families and caregivers, and represents a global economic burden (Abbott, 2011). To date, no effective pharmacological drug has been developed to reverse dementia, and the side effects of symptom alleviating drugs may not outweigh their benefits (Perneczky, 2019), therefore non-pharmacological approaches are highly needed to prevent cognitive decline and consequential dementia. Physical exercise and cognitive training have been suggested as possible strategies to protect against dementia (Livingston et al., 2020).

Most of the physical exercise and cognitive training intervention studies have been delivered in the general population. Much less is known about these effects in the population of patients with chronic diseases, in whom the disease itself and its treatment may increase the risk of cognitive decline and dementia. Patients with chronic kidney disease (CKD) are a typical example since they often suffer from hypertension, diabetes, cardiovascular diseases, and frailty that pose a risk for cognitive decline and dementia (Livingston et al., 2020; Viggiano et al., 2020). The prevalence of cognitive impairment in hemodialysis (HD) is estimated to be 30–60% (Fazekas et al., 1995; Sehgal et al., 1997; Madan et al., 2007). McAdams-Demarco et al. (2018a) found that the 10-year risk of developing dementia after starting HD is 19% in patients aged 66–70 years and increases to 28% in patients aged 76–80 years. Cognitive dysfunction also correlates with frailty in HD patients (McAdams-Demarco et al., 2015; Shen et al., 2017). A recent systematic review with meta-analysis found that the prevalence of frailty in HD patients is 46% (Lee and Son, 2021). The above findings suggest that preventive interventions are needed. Only a few studies have examined the impact of exercise programs on the preservation of cognitive function in HD patients. A 6-month home-based personalized walking exercise program in adult dialysis patients showed significant improvement in self-reported cognitive function score and quality of social interaction score compared to the control inactive group (Manfredini et al., 2017). The limitation of this study is that they used the self-reported Kidney Disease Quality of Life Short Form (KDQOL-SF), which was found in the study by Sorensen et al. (2012), as a poor indicator of neurocognitive performance in HD patients due to insufficient sensitivity. Clearly, the usage of more objective and specific measurement tools is needed.

In another study, 12 HD patients performed 3 months of tablet-based cognitive training (n-back training) during dialysis and showed improvements in Mini-Mental State Exam (MMSE) scores, Montreal Cognitive Assessment (MoCA) scores, and executive function (Noguchi et al., 2020). Limitations here were a small sample size with no control group and a significant probability of a learning effect due to the nature of used tests. McAdams-Demarco et al. (2018b) conducted a pilot study with 20 HD patients randomly assigned to a 3-month intradialytic cycling program (*n* = 6), 3-month intradialytic tablet-based brain games (*n* = 7), and a standard care control group (*n* = 7). The results showed a decrease in psychomotor speed and executive function in the control group, while the decrease was not found in the other two groups. This result is promising and it justifies the execution of larger randomized interventional studies with a better balance of confounding factors in study groups.

Considering the general lack of research in this area, the above-mentioned limitations of recent studies, aging of the CKD population and the burden of cognitive decline and frailty, further well-designed studies with non-pharmacological interventions are clearly needed. Combined cognitive and exercise training aimed at improving cognitive and executive function has been implemented and tested in the general elderly population (Colcombe et al., 2006; Levine et al., 2007; Hill et al., 2017; Jonasson et al., 2017; Butler et al., 2018) but not in HD patients. There are many unanswered questions in the dialysis population, which we aim to address in this research protocol. Therefore, the following research questions were identified:

i. What is the effect of non-pharmacological interventions in the form of combination physical exercise and cognitive training on cognitive function in HD patients?
ii. By using the combination of physical exercise and cognitive training is it possible to reduce the level of frailty in HD patients?
iii. What is the long-term effect of a short-term intervention in the form of physical exercise and cognitive training on cognitive and physical functioning in HD patients?

The study purpose is to investigate the effect of cognitive training combined with physical exercise on cognitive function, physical performance, and frailty indicators in the HD population.

## Materials and Methods

We will conduct a randomized, controlled interventional trial to examine the effects of intradialytic physical exercise in combination with intradialysis cognitive training on cognitive function, frailty indicators, and physical performance measures in population of HD patients. Main study outcome will be score on of the Test of Attentional Performance (TAP). The duration and frequency of the intervention in the form of intradialytic cycling and tablet-based cognitive training will be 12 weeks, 3 days a week. The comparator group will be HD patients under standard care, receiving only general advice on physical exercise benefits.

### Participants

Participants will be recruited from the dialysis center in University Medical Centre Ljubljana. Inclusion criteria, exclusion criteria, and withdrawal criteria for the study are listed in Table 1.

**Table 1:** Study criteria.

Inclusion criteria	Exclusion criteria	Withdrawal criteria
HD replacement therapy >3 months	Active malignant or infectious disease	Any intercurrent illness or trauma that prevents the patient from continuing the intervention for a period longer than 14 days
Over 18 years old	Uncontrolled arterial hypertension	The occurrence of an acute illness lasting longer than 3 weeks or ending less than 3 weeks before the end of the study
Stable medical condition	Angina pectoris of Canadian Cardiovascular Society grade 2–4	Diagnosis of a malignant disease throughout the research
Able to walk independently	New York Heart Association heart failure grade 3 or 4	Cerebrovascular or other cardiovascular event (new-onset angina pectoris, myocardial infarction, symptomatic peripheral arterial obliterative disease, heart failure hospitalization)
	Severe cognitive impairment and dementia	Withdrawal of consent to participate
	History of limb amputation	
	Any other condition that could cause the patient to be clinically unstable	

HD, hemodialysis.

The study will be conducted in accordance with the ethical standards of the 1964 Declaration of Helsinki and has been approved by the Slovenian Medical Ethical Committee (number 0120-474/2021/4). Participants will sign an informed consent form before participating in the study. The study was registered at ClinicalTrials.gov under NCT05150444.

### Procedures

Throughout the experiment, testing procedures will be conducted in the same facility, by the same researchers, with the same equipment at a similar time of the day. Testing will be performed on non-dialysis day. Finally, the risks and benefits will be explained to each participant prior to enrollment in the study. The risks of study participation are associated with possible deterioration in health status due to physical exertion. However, there will be adequate rest between each physical performance test and physical exertion during dialysis will be adapted to individual abilities with continuous monitoring of hemodynamic status and subjective exertion throughout the dialysis procedure. There will be full coverage with physician presence in research facilities.

#### Study Interventions and Protocol

After the screening, recruitment, and baseline measurements, the patients will be randomized using sealed envelopes in 1:1 ratio into two groups:

– combined cognitive and exercise training (EXP) group and
– control (CON) group

The EXP group will first exercise during dialysis (three times a week; 12 weeks) for ~30 min on a customized ergometer. They will start with a 3-min warm-up, and then, the resistance will be implied to each individual according to the rate of perceived exertion of 4th to 5th grade on a 10-grade Borg scale (Bogataj et al., 2020). After a break, they will be given tablets in order to play »cognitive stimulation games« on a CogniFit platform (~30–45 min). CogniFit “brain training” requires patients to repeatedly solve cognitively challenging tasks that target specific cognitive areas. These cognitive tasks are presented in the form of colorful, visually appealing “mini-games” to promote fun and enjoyment, thereby increasing motivation and engagement. The CogniFit platform offers a wide range of games (e.g., Jigsaw, Mandala, Sudoku, Puzzles, Word Quest, Piece Making, Tennis Bowling, and Line Changer, etc.). The difficulty level of the “brain exercises” automatically adjusts to the patient’s abilities as they practice and train. The cognitive areas targeted are: memory, reasoning, coordination, and attention with their subcategories. The training will be performed by a qualified kinesiologists (a professional coach experienced in prescribing and guiding the intradialytic exercise) and by a clinical psychologists with experience in cognitive testing of chronic patients. The CON group will receive standard HD care.

**Figure 1:**
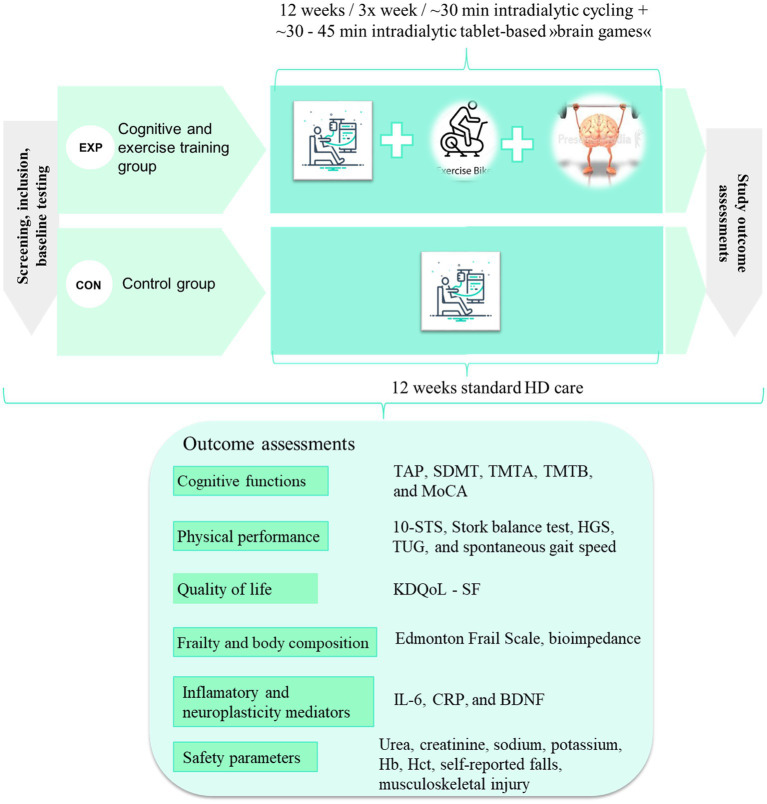
Proposed design of the study and proposed outcome measures. EXP, experimental group; CON, control group; TAP, Test of Attentional Performance; SDMT, Symbol Digit Modalities Test; TMTA, Trail Making Test A; TMTB, Trail Making Test B; MoCA, Montreal Cognitive Assessment; 10-STS, 10 repetition sit-to-stand test; HGS, handgrip strength; TUG, Timedup and Go test; KDQoL-SF, Kidney Disease Quality of Life Short Form; BDNF, Brain-Derived Neurotrophic Factor; IL-6, Interleukin-6; hs-CRP, high-sensitivity C-reactive protein; Hb, hemoglobin; Hct, hematocrit.

Graphically we present the study design in Figure 1. We plan to repeat the measurements 6 months after the end of the intervention to measure the longer-term retention effect of intervention.

#### Primary Study End Point

##### Cognitive Performance (Neuropsychological Assessment Battery)

Cognitive functions will be assessed with tests previously used in HD patients (Costa et al., 2014; San et al., 2017; Zimmermann and Fimm, 2017b; Jafari et al., 2020), including Montreal Cognitive Assessment (MoCA), Trail Making Test A and B (TMTA and TMTB), Symbol Digit Modalities Test (SDMT), and computerized Test of Attentional Performance (TAP).

TAP score is selected as the main outcome of this study. The rationale for the selection of the main outcome is: (i) absence of significant level of test-related learning effect (Zimmermann and Fimm, 2017b), (ii) sensitive to effects of physical exercise (Noguera et al., 2019), and (iii) attention is one of the most affected cognitive domains in dialysis patients (Dixit et al., 2013). From the TAP test battery, we will include subtests Alertness, Selective attention, Divided attention, and Sustained attention. Low to moderate training effects were reported for the TAP subtests (Zimmermann and Fimm, 2017a). The authors stated that performance remained stable in most of the TAP tasks at most of the testing occasions, suggesting that the performance in these tasks is robust and unaffected by learning effects or task repetition harassment.

MoCA (Nasreddine et al., 2005) is a global cognitive function assessment tool covering eight cognitive domains. The psychomotor speed and executive function will be measured by TMTA and TMTB (Corrigan and Hinkeldey, 1987). The SDMT will be used to assess the psychomotor speed, efficiency of information processing, ability to switch between mental sets of the information and to maintain and manipulate information in working memory (Smith, 1982; Benedict et al., 2017). It is a reliable and valid test for assessing information processing speed, efficiency and executive functioning domains (Benedict et al., 2017).

#### Secondary Study End Points

##### Physical Performance

We will test the patients with selected functional performance tests [10 repetition sit-to-stand test (10-STS), stork balance test, handgrip strength test (HGS)], exact methods already described in our previous study (Bogataj et al., 2020), with Timed Up and Go (TUG) test (Richardson, 1991; Ortega-Pérez de Villar et al., 2018), and spontaneous gait speed (Bučar Pajek et al., 2016).

##### Frailty Assessment

Frailty indicator will be assessed by the Edmonton Frail Scale (Rolfson et al., 2006; Garcia-Canton et al., 2019). The Edmonton Frail Scale assesses nine subscales: cognition, general health, functional independence, social support, medication use, nutrition, mood, continence, and functional performance in 11 items. The highest score is 17 points and correlates with the highest level of frailty (Rolfson et al., 2006). A score of 0–4 points represents no frailty, a score of 5–6 represents vulnerability, a score of 7–8 represents low frailty, a score of 9 to 10 represents moderate frailty, and a score above 11 represents severe frailty (Aygör et al., 2018).

##### Body Composition

Phase angle, fat, and lean body mass will be assessed with bioimpedance analysis (BCM Fresenius Medical Care).

##### Quality of Life

The participants’ quality of life will be assessed with validated Kidney Disease Quality of Life – Short Form questionnaire (Korevaar et al., 2002).

##### Blood Sampling and Potential Biomarkers (Biochemical Analysis)

The concentrations of the brain-derived neurotrophic factor (BDNF) will also be measured to explore the possible mechanisms underlying the effects of exercise on cognitive function. It was demonstrated that BDNF in the HD patients was significantly lower when compared to the age-matched control group (Zoladz et al., 2012). We decided to include BDNF biomarker in our analysis based on findings of Nofuji et al. (2012), reporting that the BDNF concentration was found to significantly change with physical exercise. Furthermore, a recent study showed that BDNF is associated with decreased physical performance and the prevalence of severe sarcopenia and frailty in HD patients (Miyazaki et al., 2021). Blood sampling will be performed by our staff (qualified HD nurses). Blood samples (∼7 ml) will be drawn *via* arteriovenous fistula before initiating HD procedure. Blood analysis will also include variables relevant to discuss the inflammation and HD-related laboratory parameters (such as CRP, urea, IL-6, and electrolytes).

##### Safety Parameters

The safety parameters assessed will be urea, creatinine, sodium, potassium, hemoglobin, hematocrit, self-reported falls, and musculoskeletal injuries.

### Statistical Methods Including Sample Size Calculation

The primary outcome of this study will be the cognitive function assessed with the subtest “Alertness” of the Test for Attentional Performance (TAP, Version 2.3; Psytest, 2012).

The sample size was calculated using G*Power software (version 3.1; Faul et al., 2009) on the basis of the results of the study by Briken et al. (2014). For the calculation, the scores of the subtest alertness from the TAP test battery which were obtained from the group that completed bicycle ergometry before (376.91 ± 151.67) and after (302.64 ± 83.09) the treatment were used. The alpha error probability was set to 0.05, the 1-beta error probability to 0.80, while the effect size was taken from the previously mentioned research (0.314). A sample size of 22 participants was calculated. Allowing for a 20% attrition rate, a total number of 27 participants is required, with 14 participants assigned to each group. Analyses will be conducted according to the intention-to-treat principle.

SPSS 24.0 (SPSS, Inc., Chicago, IL, United States) software will be used for all calculations. All data will be presented with mean ± standard deviation and 95% confidence intervals when appropriate. Normality will be confirmed by using the Shapiro–Wilk test, with additional Q–Q plot visual inspection. Independent-sample *t* test, *χ*^2^, or Mann–Whitney tests will be used to determine group differences in clinical and demographic variables, depending on the comparison and test assumptions. The main effects will be analyzed using a mixed general linear model (GLM), taking into account the groups (EXP and CON) and time (baseline and after 12 weeks) as factors. After determining the interaction effect, a secondary analysis will be used to determine the time effect in both groups. Additionally, the degree of effect will be determined for dependent variables by using partial eta-squared (*η*^2^). Partial eta squared readings of 0.02, 0.13, and 0.33 were rated differences as small, moderate, and high (Pierce et al., 2004). Furthermore, in the case of unmatched baseline means, the analysis of covariance (ANCOVA) with baseline measurements entered as a covariate will be applied. For non-parametric data, a Friedman ANOVA will be applied, followed by a Sign-test separately for each scale. Statistical significance will be set at values of *p* < 0.05.

## Discussion

The presented study will be the first randomized controlled intervention trial combining physical and cognitive exercise in HD patients. By testing the possible beneficial effect of non-pharmacological interventions it will address a significant unmet need of dialysis patients. The foreseen achievements are to identify the benefits of combined physical exercise and cognitive training. We hypothesize that the experimental group will improve significantly and to a clinically meaningful effect size in cognitive and physical domains after the intervention compared to the control group. We will use novel instrumentation for sensitive detection of cognitive adaptation.

To the best of our knowledge, MMSE, MoCA, and Modified Mini-Mental State examination (3MS) are the most commonly used tests for cognitive screening in studies investigating the effects of different interventions on cognition in HD patients. Among the aforementioned instruments, MoCA has been shown to be the most suitable instrument for cognitive screening in the HD population with good sensitivity and specificity (Lee et al., 2018). However, the tests mentioned above are mostly used as screening tests for mild cognitive impairment and dementia and are not sensitive enough to detect intervention effects (Dong et al., 2010; Sheehan, 2012). Furthermore, all these tests are subject to learning effects, which limits the internal validity of prior studies. Accordingly, future studies should focus on selecting more specific and sensitive tests rather than using global/general cognitive tests and use the tests with low learning effect bias.

Combined cognitive and physical exercise training over a 3-month period improved executive function in older adults and was more effective than cognitive training or exercise training alone (Fabre et al., 2002; Anderson-Hanley et al., 2012). Moreover, in the pilot study (McAdams-Demarco et al., 2018b), cognitive training and exercise training performed separately were able to prevent decline in executive functions and psychomotor speed in HD patients. The results from a these limited studies in HD patients may support the hypothesis of a positive effect of cognitive and exercise training on cognitive function; however, a much better and clearer study design, especially to prevent the bias from learning effects and to secure adequate statistical power, is needed. Suppose there truly is a positive effect of physical exercise and/or cognitive training on cognitive function in dialysis patients, it is plausible that the strongest effects may be found with a combination of both interventions.

With this in mind, it is a reasonable step to combine the use of physical exercise and cognitive training with the aim of improving the functional (cognitive and physical) status of HD patients. HD procedure is a unique opportunity for the implementation of these types of combined interventions. In our research protocol, we address the unanswered questions by implementing the combined intervention and using more sensitive low learning effect cognitive tests. This research will also demonstrate the feasibility of using the innovative cognitive platform to apply cognitive training to HD patients. Ultimately, the basic knowledge gained can be used to develop appropriate interventions to mitigate the cognitive decline and maladaptation caused by physical inactivity. Based on our findings, we could develop guidelines and exercise protocols (physical and cognitive) aimed at improving cognitive and physical performance, thus improving the quality of life of HD patients. Importantly, in case our hypotheses will be confirmed, we will be able to offer evidence-based improvement of chronic renal replacement therapy programs.

## Ethics Statement

The studies involving human participants were reviewed and approved by Slovenian Medical Ethical Committee. The patients/participants provided their written informed consent to participate in this study.

## Author Contributions

ŠB and JP conceptualized the study design. ŠB drafted the manuscript. JP, NT, and MP reviewed the manuscript. All authors have read and approved the final version of the manuscript.

## Funding

This research is funded by the Slovenian Research Agency (postdoctoral research project Z3-3212) and the ARRS research and infrastructure program number P3-0323 (Renal diseases and renal replacement therapy).

## Conflict of Interest

The authors declare that the research was conducted in the absence of any commercial or financial relationships that could be construed as a potential conflict of interest.

## Publisher’s Note

All claims expressed in this article are solely those of the authors and do not necessarily represent those of their affiliated organizations, or those of the publisher, the editors and the reviewers. Any product that may be evaluated in this article, or claim that may be made by its manufacturer, is not guaranteed or endorsed by the publisher.
